# Multi-site benchmarking of clinical ^13^C RF coils at 3T

**DOI:** 10.1016/j.jmr.2020.106798

**Published:** 2020-09

**Authors:** Juan Diego Sánchez-Heredia, Rie B. Olin, Mary A. McLean, Christoffer Laustsen, Adam E. Hansen, Lars G. Hanson, Jan Henrik Ardenkjær-Larsen

**Affiliations:** a Department of Health Technology, Technical University of Denmark, Kgs. Lyngby, Denmark; b Cancer Research UK Cambridge Institute, University of Cambridge, Cambridge, UK; c MR Research Centre, Department of Clinical Medicine, Aarhus University, Aarhus, Denmark; d Department of Clinical Physiology, Nuclear Medicine and PET, Rigshospitalet, University of Copenhagen, Copenhagen, Denmark; e Danish Research Centre for Magnetic Resonance, Centre for Functional and Diagnostic Imaging and Research, Copenhagen University Hospital Hvidovre, Denmark; f GE Healthcare, Brøndby, Denmark

**Keywords:** RF coil, SNR, ^13^C MRI, Hyperpolarization, Quality assurance, Parallel imaging

## Abstract

A quality assurance protocol for RF coils is proposed, which can be used for volume (Tx/Rx) and surface (Rx) coils. Following this protocol, a benchmarking of seven coils (from three different MR sites) dedicated to ^13^C MRI at 3T is reported. Coil performance is particularly important for 3T MRI at the ^13^C frequency, since the coil-to-sample noise ratio is typically high. The coils are evaluated experimentally using the proposed protocol based on MR spectroscopic imaging performed with two different phantoms: one head-shaped, and one with cylindrical shape and nearly twice the volume of the first one. To achieve an unbiased SNR comparison of volume and array coils, coil combination was done using sensitivity profiles extracted from the data. SNR, noise correlation matrices and example g-factor maps are reported. For globally calibrated, equal excitation angles, the measured SNR shows large differences for the volume coils of up to 115% at the phantom center for a head phantom. The arrays show lower differences in superficial SNR. The sample surface depth at which the volume coils outperform the arrays is estimated to 7 cm, and SNR furthest away from the coil surface is 28% lower for the best array compared to the best volume coil. A broad set of coils for ^13^C at 3T have been benchmarked. The results reported, and the method used to benchmark them, should guide the ^13^C community to choose the most suitable coil for a given experiment.

## Highlights

• A quality assurance (QA) protocol for ^13^C RF coils is proposed.
• The protocol allows SNR comparisons between volume and surface coils.
• Seven different ^13^C coils are evaluated across platforms and MR sites.
• The results show the importance of careful QA for ^13^C coils at 3T.
• All data and source code of the proposed protocol are made publicly available.

## Graphical abstract

**Figure:**
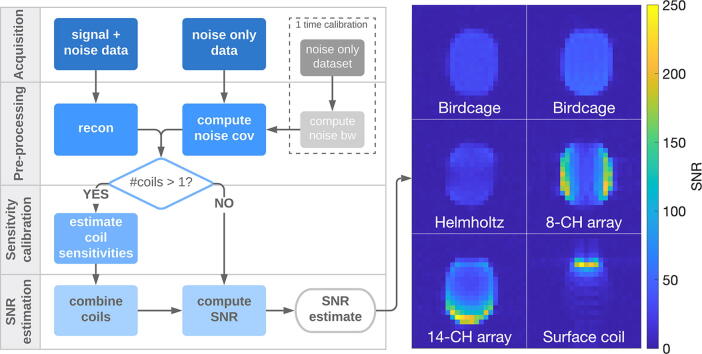

## 1 Introduction

The number of magnetic resonance (MR) applications for hyperpolarized ^13^C compounds using dissolution dynamic nuclear polarization (dDNP) [1], [2], [3] has grown steadily over the last decade. One of the challenges of this technique for its clinical use is the need of dedicated radiofrequency (RF) hardware for the lower Larmor frequency of ^13^C, as opposed to the standard ^1^H RF hardware. Apart from its cost, transmit amplifiers do not imply a large technological burden, while the RF coils pose a significant challenge due to the lower resonance frequency of the ^13^C nuclei and combination with existing ^1^H coils. In practice, the lower frequency implies that the coils operate in (or close to) a regime where their electronic noise is not negligible [4]. Thus, relatively small differences in coil losses will have a direct impact on the signal-to-noise ratio (SNR) of the MR images. At higher frequencies, the dominating sample noise will often mask the effect of coil conductor losses on SNR.

Standard clinical ^1^H receive coils are often array coils as these allow accelerated acquisition through parallel imaging [5]. For hyperpolarized MR, the use of arrays might be particularly encouraged, as SNR for parallel imaging in this case is not penalized (ideally) by the reduced acquisition time, but only by the geometry factor (g-factor) [6], [7], explained by the finite, non-recoverable nature of the magnetization of the hyperpolarized nuclear spins. This has triggered the development of dedicated ^13^C arrays aimed at parallel imaging [8]. However, the question of optimal coil geometry in terms of SNR remains open, and also that of SNR performance for ^13^C arrays under practical conditions compared to simpler volume coils.

The purpose of this study is twofold: to propose a new acquisition and reconstruction protocol for quality assurance, and to evaluate and compare different ^13^C coils across platforms and MR sites.

The comparison is based on six different ^13^C coils of different volume and surface array geometries covering a similar field of view (FOV), and an additional receive-only surface loop (used as a reference for superficial SNR). While other studies have been done based on numerical simulations [9], the goal here is to study how the different designs perform in practice. The coils include two home-built coils and five commercial coils. Three of the coils are transmit-receive volume coils and four are receive-only coils, with four of the coils designed for head imaging. With this selection, we choose to put special focus on ^13^C human brain imaging, a challenging clinical case, which has attracted much interest lately. In fact, the first studies of hyperpolarized [1-^13^C]pyruvate and [2-^13^C]pyruvate in human brain have already been presented [10], [11], [12], [13] with results suggesting a need for tailored receive coils.

Another focus of this study, in context of the proposed quality assurance (QA) protocol, is coil combination for array coils to provide a fair comparison of array and volume coils. If coil combination is not done optimally, an SNR bias dependent on the number of elements will obscure the comparison between different arrays and volume coils [14]. Furthermore, how array data are combined has shown to be critical for hyperpolarized experiments [15], where the dynamically acquired data usually have low SNR.

The results presented in this study were obtained at three different MRI systems: a GE Discovery MR750 scanner (GE Healthcare, Waukesha, WI, USA) at Aarhus University Hospital (Aarhus, Denmark), a Siemens Biograph mMR PET/MR scanner (Siemens Healthcare, Erlangen, Germany) at Rigshospitalet (Copenhagen, Denmark), and a GE Discovery MR750 scanner at Addenbrooke's Hospital (Cambridge, UK). With these representative configurations, the presented methods and results shed light on the optimal use of RF coils for ^13^C MRI at 3T.

## 2 Methods

### 2.1 RF coils

The SNR factor related to the RF coil sensitivity of a given MR experiment can be represented as Eq. (1):

(1) $$S_{\mathrm{RF}}\approx \frac{\omega (B_{1}/I)}{\sqrt{4k_{B}R_{\mathrm{eq}}T_{\mathrm{eq}}}}$$

The factor B_1_/I is the local magnetic coupling between coil and sample. For a receive coil, (using the reciprocity principle) it can be calculated as the B_1_ field that an equivalent transmit coil would generated across the sample for a unit of current. The other term related to the coil is the factor R_eq_·T_eq_, which represent the total equivalent resistance (temperature averaged) induced in the coil. This includes both coil, sample and preamplifier noise components. Additionally, ω is the angular B_1_ frequency, and k_B_ is the Boltzmann’s constant.

In that equation, the most relevant term for comparing different coils is B_1_/I, which gives a measure of the local receive sensitivity. For voxels close to the coil, this term is notably higher for surface coils than for volume coils. Through the use of coil arrays and appropriate coil combination, this superior B_1_/I provided by surface coils can be extended to big FOVs, which explains their widespread use.

The other term that is coil-dependent is Req. Ideally, the equivalent resistance of an MR experiment would be sample-dominated, meaning that most of the noise present in the experiment would come from the ionic currents in the conductive biological sample. However, at low frequencies, the electrical loss of the RF coil cannot be neglected. This term is also very different in surface coils and volume coils, and therefore at lower Larmor frequencies (e.g. of ^13^C), SNR performance differences due to this factor are more likely.

**Fig. 1:**
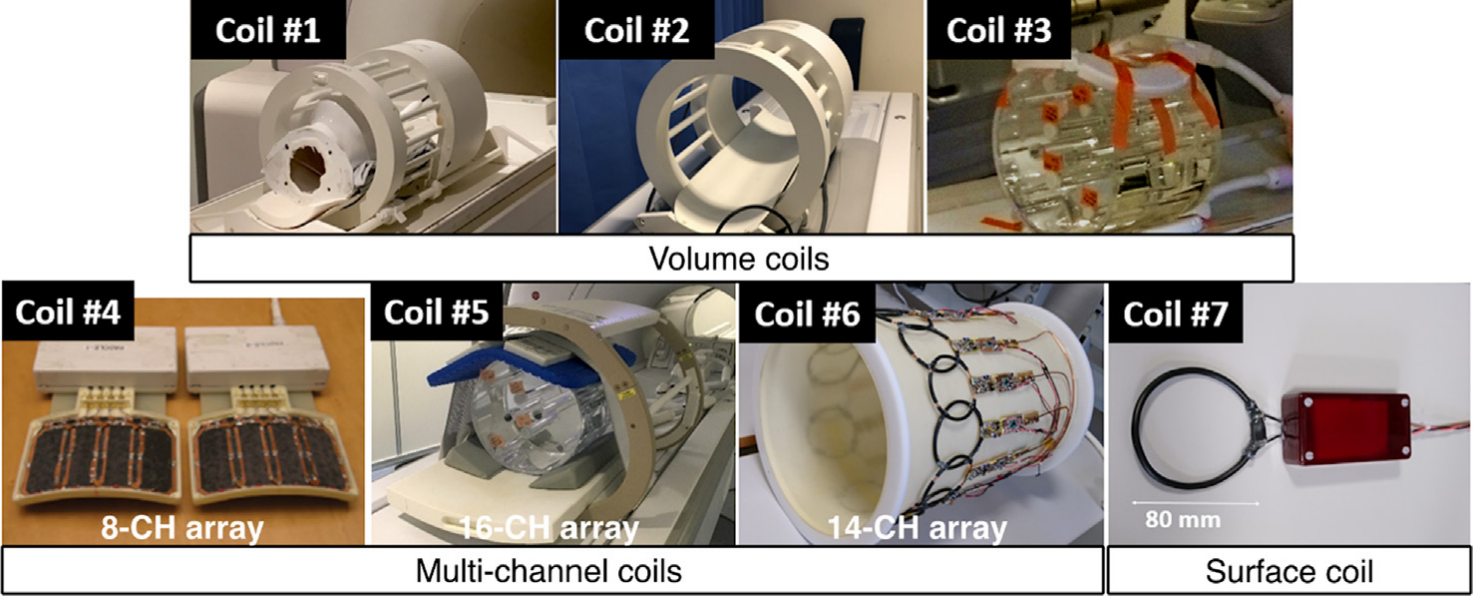
The different RF coils included in the benchmarking.

In this context, seven different RF coil setups were evaluated as listed in Table 1. Photos of the coils are shown in Fig. 1. This selection includes three transmit-receive volume coils, three receive-only arrays and one receive-only single loop.

**Table 1:** Description of the evaluated coils.

	Coil Parameters				MR System	
	Type	Geometry	T/R Mode	Manufacturer	Brand	Location
Coil #1	Volume	Birdcage	Tx-Rx	RAPID Gmbh	Siemens	Copenhagen, DK
Coil #2	Volume	Birdcage	Tx-Rx	RAPID Gmbh	GE	Cambridge, UK
Coil #3	Volume	Helmholtz	Tx-Rx	Pulsteq	GE	Aarhus, DK
Coil #4	Surface	8-CH Array	Rx-Only	GE	GE	Aarhus, DK
Coil #5	Surface	16-CH Array	Rx-Only	RAPID Gmbh	GE	Aarhus, DK
Coil #6	Surface	14-CH Array	Rx-Only	HYPERMAG	GE	Aarhus, DK
Coil #7	Surface	Single Loop	Rx-Only	HYPERMAG	GE	Aarhus, DK

**Coils #1 and #2** are birdcage coils designed for human head imaging and used for both transmit and receive. Coil #1 has an inner diameter of 265 mm and a length of 210 mm, while Coil #2 has an inner diameter of 255 mm and length of 170 mm. Both are quadrature coils. Coil #1 is ^13^C dedicated (single-tuned), while Coil #2 is dual-tuned to ^1^H and ^13^C. The third volume coil, **Coil #3**, is a flexible Helmholtz pair also used in transceiver mode. The loops forming the Helmholtz pair have 200 mm diameter and variable separation.

**Coil #4** is a commercial receive-only array (GE Healthcare, Waukesha, WI, USA) that consists of 8 elements, each being a rectangular loop of 50x100 mm^2^, in a rigid frame [7], [16]. The 8 elements are arranged in two separate paddles of 4. While this array was designed originally for human head imaging, its flexibility in positioning makes it useful for other imaging cases as well, and it is in practice used as one of the standard multipurpose coils on GE systems.

**Coil #5** is a commercial 16-channel receive-only array (RAPID Gmbh, Rimpar, Germany), made of rectangular loops of approximately 60x130 mm^2^, with a similar design to [17]. The array is divided in two separate modules: a rigid module placed below the sample and a flexible module placed above the sample. Both modules have a 2x4 layout of 8 loops each. This array was originally designed for human cardiac imaging, but can also accommodate other sample geometries due to the flexibility of its top module.

**Coil #6** is a home-built 14-channel receive-only array, made of circular loops with 80 mm diameter. The coils are placed around a cylinder with 250 mm diameter; a suitable size for human head imaging. Each loop is critically overlapped with its neighbors, while decoupling to the next-neighboring coils is provided by the preamplifiers as described in [18], [19].

With these selections, we aimed to include coils that provide the best (or close to optimal) SNR at both the center of the considered FOV and at the surface. The inclusion of **Coil #7** (single loop with 80 mm diameter, receive-only) in this study provides a comparison for surface SNR, which is not impaired by mutual coupling to other elements (as is the case in an array). For Coils #6 and #7, simulated unloaded and loaded Q-factors [40] and B1- field maps, are included in the supplementary material. Simulations were carried out using CST (Darmstad, Germany). Both coils were simulated without loading, with a cylindrical load of 250 mm diameter and 20 mm length (tissue properties: ε_R_ = 34, σ = 0.47 S/m [20]), and with a head model of a human adult female (ELLA, Virtual Family V1, ITIS Foundation, Switzerland [21]). All receive-only coils were used in combination with a separate ^13^C transmit coil of the clamshell type (RAPID Gmbh, Rimpar, Germany). The coil, similar to the one described in [22], [23], measures 400 mm in diameter and 300 mm in length.

### 2.2 Phantoms

Two different phantoms were used for characterization of the coils described above. The first one is a Specific Anthropomorphic Mannequin (SAM) phantom (IXB-030, IndexSAR, Surrey, UK): a head-shaped phantom with a 2-mm thick shell, measuring 300 mm from top to bottom, and 225 mm in maximum outer perimeter. The phantom was filled with a solution of ethylene glycol (99.8%; natural abundance ^13^C; triplet with J_CH_ of ~142 Hz) (Sigma Aldrich, Missouri, US) and NaCl (17 g/L) for loading. The concentration of NaCl was adjusted to match muscle tissue conductivity at 32 MHz (0.66 S/m [20]) and estimated using an RF dielectric probe kit (85070E, Keysight, CA, USA). The measured relative permittivity was 38.7, which agrees well with literature [24]. The T_1_ relaxation time constant of the ethylene glycol solution at room temperature and 3T was measured to be ~0.7 s.

**Fig. 2:**
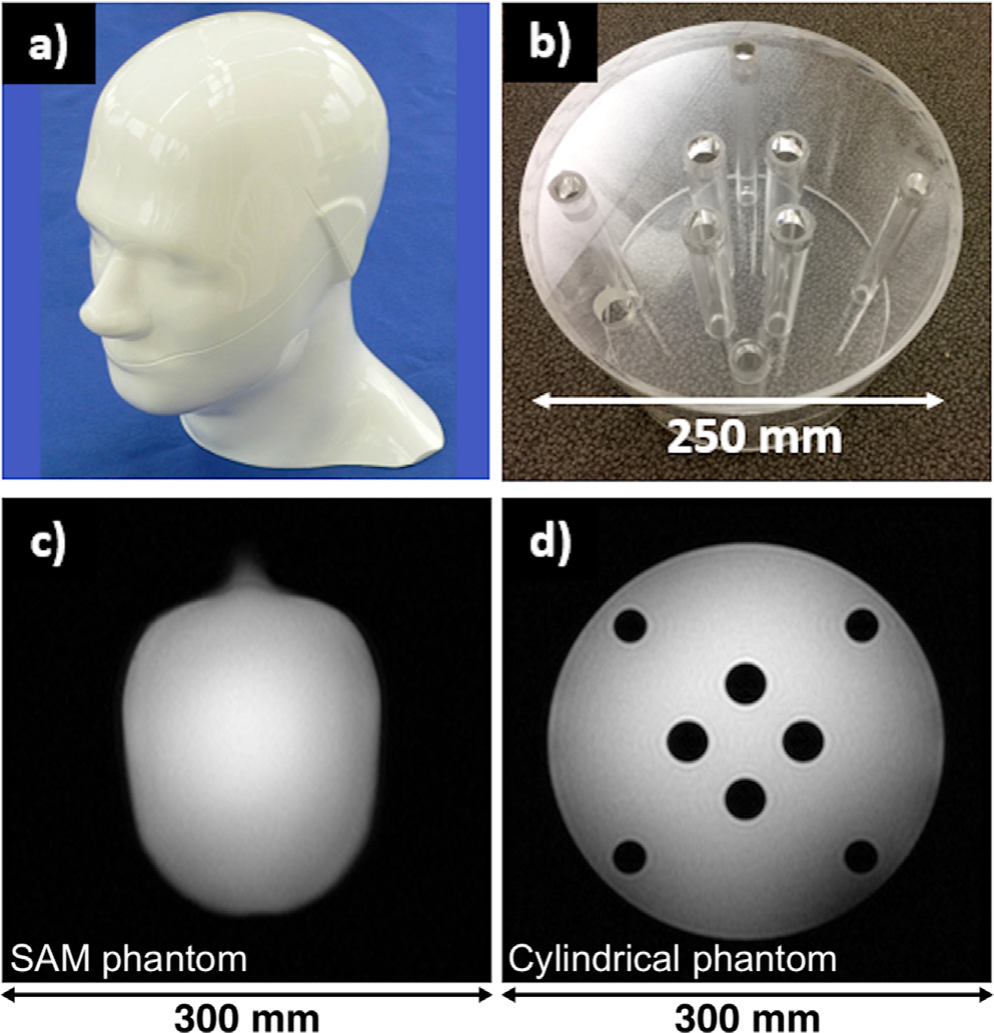
The two phantoms used in this study: (a) SAM phantom, (b) cylindrical phantom. ^1^H MR images of an axial slice with FOV 30 × 30 cm^2^ are shown for the phantoms in (c) and (d).

The second phantom is a cylinder of 200 mm in length with an outer diameter of 250 mm. The cylinder is made of acrylic plastic (polymethylmethacrylate – PMMA) with a shell thickness of 5 mm, and was filled with an ethylene glycol solution identical to the one used for the SAM phantom. The cylindrical phantom has eight sealed internal compartments that can be filled with additional compounds (not used for this study). The cylindrical phantom was used to more accurately characterize surface SNR for the different coils, due to its axial slice symmetry (contrary to the SAM phantom). Both phantoms are shown in Fig. 2.

Both phantoms were used to characterize each of the coils described above, with two exceptions: Coil #2 could not be measured with the cylindrical phantom in Cambridge (UK), due to practical challenges. Coil #5 was not measured with the SAM phantom, as we did not consider it appropriate to characterize an array designed for cardiac imaging with a head phantom (a very unlikely use of it). Therefore, the final comparison includes measurements from six of the coils for each phantom: Coils #1, #2, #3, #4, #6 and #7 with the SAM phantom, and Coils #1, #3, #4, #5, #6 and #7 with the cylindrical phantom.

### 2.3 Quality assurance protocol – MR acquisition

**Fig. 3:**
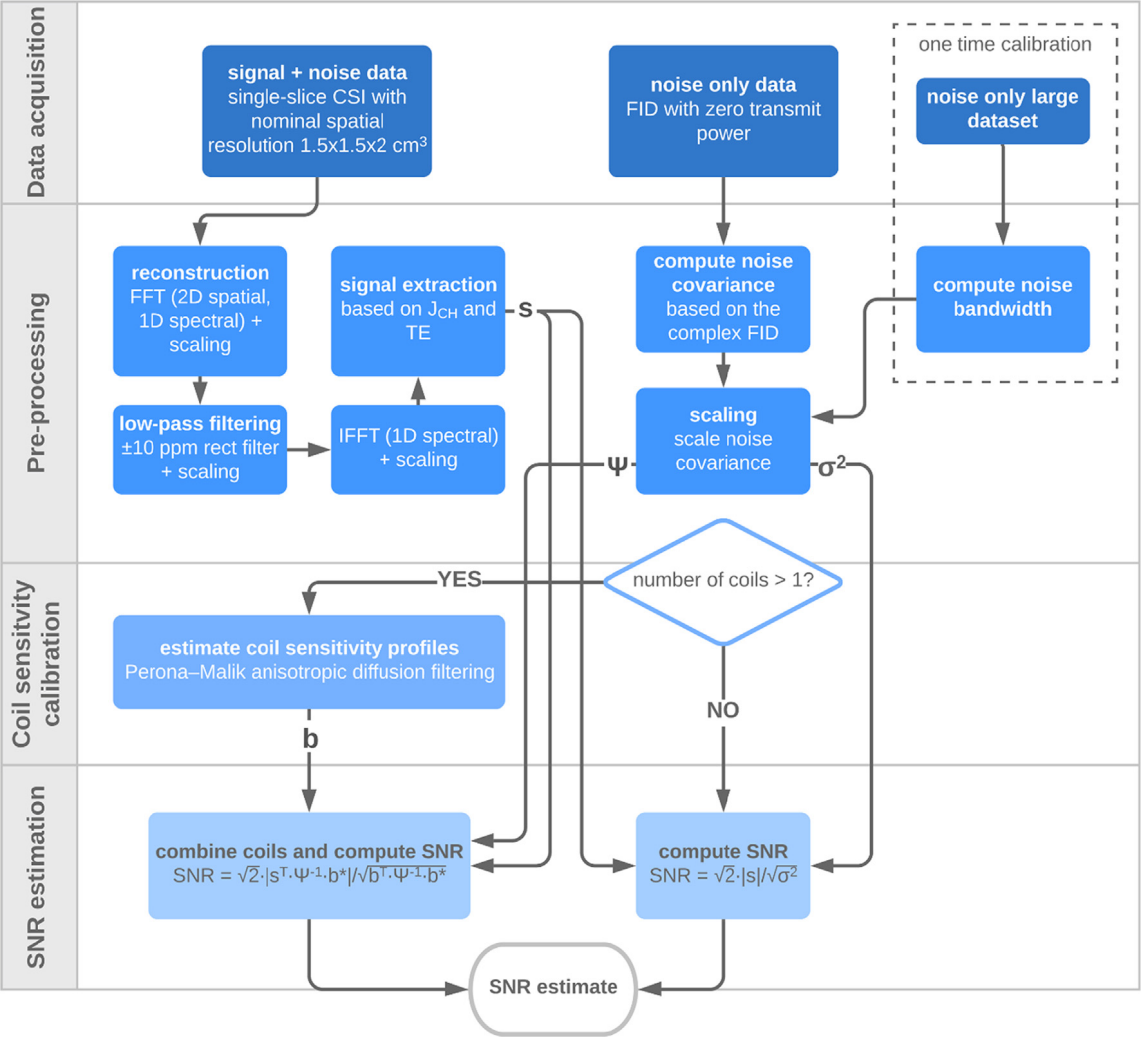
Summary of the quality assurance protocol. “s” refers to the extracted signal estimate, “σ^2^” to the scaled noise variance, “Ψ” to the scaled noise covariance matrix, and “b” to the estimated coil sensitivity profiles.

To estimate SNR for the coils and phantoms described above, a QA protocol was established based on methods described in [25]. The full protocol from acquisition to final SNR computation is summarized in the flowchart in Fig. 3. Additional details are given in this section and the following.

In the protocol, MR acquisition is done using a non-refocused chemical shift imaging (CSI) sequence with the center frequency at the ethylene glycol center peak resonance. The CSI sequence was chosen due to its robustness and availability in most scanner’s standard software packages, including necessary raw data access. The sequence was scanned in the axial plane with a FOV of 36x36 cm^2^, a slice thickness of 20 mm, and matrix size of 24x24. The nominal spatial resolution is therefore 1.5x1.5x2 cm^3^. Other sequence parameters were: soft pulse excitation with nominal flip angle = 70°, TR = 1000 ms, spectral bandwidth = 5000 Hz, FID points = 1024. The used RF pulse was a standard 1.8-ms minimum-phase (2289-Hz bandwidth) for the GE system and a 0.5-ms linear-phase (3000-Hz bandwidth) for the Siemens system. The sequence was run with minimum echo time (time until sampling), which was 2.7–3.1 ms for the GE systems and 2.3 ms for the Siemens system. The total acquisition time amounts to 9 min 36 s.

RF power calibration prior to CSI acquisition was done slightly differently for the different scanner vendors. For the Siemens system, multiple FIDs were acquired at different RF power settings for external sinusoidal fitting to find the 90° reference voltage [26]. For the GE systems, an automated Bloch–Siegert phase shift method was used as described in [27]. Both calibrations used non-localized excitation and included determination of the center frequency, manually for the Siemens system and automatically for the GE system. A non-localized calibration is not optimal, especially when using superficial coils, but we intent to keep the proposed protocol such that is valid for any possible kind of coil geometry and target FOV.

A separate measurement was done to estimate the noise for the subsequent SNR evaluation. The noise measurement was acquired as a non-localized FID with TR = 1000 ms, spectral bandwidth = 5000 Hz, FID points = 1024 and flip angle = 0°, i.e. with zero transmit power (ia_rf1 = 0 for the GE systems). To account for the fact that the raw noise spectrum is not flat across the full bandwidth, a scanner-dependent noise equivalent bandwidth factor needs to be estimated [25]. This was done for each scanner based on a one time noise-only measurement and calculations as described in [25]. For the GE scanners, the bandwidth factor was estimated at 0.845. For the Siemens scanner, the bandwidth factor was estimated at 0.793.

All measurements were done following standard automatic shimming at the proton frequency.

Additional to the data acquisition stated in the QA protocol, transmit maps for Coil #2 and the clamshell coil used in combination with Coils #4–7 were also measured. Transmit map measurements were done using the Bloch–Siegert approach described in [28] by repeating the CSI acquisition described above twice with a positive and negative offset frequency of the Bloch–Siegert pulse, respectively. Both transmit maps were acquired with the SAM phantom. The transmit map for the clamshell coil was acquired in combination with Coil #4. The transmit maps were smoothed using first-order local polynomial fitting with a region size of 7.5 cm.

### 2.4 Reconstruction and SNR estimation

Siemens raw data were read using source code from the FID-A toolkit [29]. Image reconstruction of the raw CSI data was initiated by Fourier transformation along the spatial and spectral dimensions without any apodization and zero-filling. Next, all spectra were low-pass filtered by multiplication with a rectangular function spanning ±10 ppm and including all signal, and were then Fourier transformed back to the temporal domain. During all processing steps, the data were scaled appropriately with respect to the Fourier transform and filter such that the input and output had the same noise standard deviation [25]. Signal data extraction was done based on the first in-phase time point of the FIDs, instead of peak amplitude, to minimize signal variations from shimming and resulting spectral linewidths. The first in-phase time point within the sampling period was determined based on the J-coupling and the echo time. The ethylene glycol J-coupling was experimentally estimated from the peak-to-peak distance in a high signal voxel. The first in-phase time point was found at 4.4 ms on average for the data acquired in this study.

The complex FID from the separate noise measurement was used to estimate the coil noise covariance matrix, which for a single-receiver coil is a scalar representing the noise variance. The covariance matrix was subsequently scaled with respect to the scanner-dependent noise bandwidth.

At this point, both signal and noise data have been pre-processed for the SNR estimation. However, to assure a fair comparison between coils with different numbers of receive elements, the next step of the SNR estimation, involving coil combination for arrays, needs to be done with extra care. When array data are combined by sum-of-squares, the probability distribution of the combined magnitude image follows a non-central chi distribution [14]. This depends on the number of coil elements, and noise statistics will therefore vary for arrays with different number of elements following sum-of-squares combination. Alternatively, when coil combination is done optimally with complex data and using the coil sensitivity profiles as weight functions as defined by Roemer et al. [30], the SNR statistics of the combined magnitude image follow those of a single-receiver system; a Rician distribution. Sensitivity-based coil combination of array data therefore provide an even starting point when comparing SNRs of arrays and volume coils.

The coil sensitivity profiles of the included arrays were not known a priori but could be estimated based on the reconstructed complex CSI data as the close to uniform phantom coil images represent a good, but noisy estimate of the profiles themselves. The sensitivity profiles were extracted from the data by performing edge-preserving smoothing using Perona–Malik Anisotropic Diffusion filtering [31]. The degree of smoothing and anisotropy of the filter were set relative to the SNR level on an element-by-element basis, such that a high maximum SNR at the object edge would result in high anisotropy, and a high overall SNR would result in minimal smoothing and vice versa.

The final SNR images were estimated by Eq. (2)

(2) $$\mathrm{SN}R_{n=1}=\sqrt{2}\left|s\right|/\sqrt{\sigma^{2}}$$

for single-receivers (with number of coil elements, *n*, equal to one), in which *s* is the signal from the CSI data and σ^2^ is the scaled noise variance. For array coils, the SNR images were estimated by Eq. (3)

(3) $$\mathrm{SN}R_{n>1}=\sqrt{2}\left|s^{T}Â\cdot \Psi^{-1}Â\cdot b^{*}\right|/\sqrt{b^{T}Â\cdot \Psi^{-1}{Â\cdot b}^{*}},$$

which include sensitivity-based coil combination with *b* representing the estimated complex sensitivity profiles. The expression accounts for coil noise correlations with Ψ representing the scaled noise covariance matrix. The factor of $\sqrt{2}$ was included as in [32] to account for the SNR definition in terms of the real channel noise component.

To evaluate variations in field conditions across experiments, T_2_* relaxation times were estimated based on the inverse of the full width at half maximum for the ethylene glycol center peak.

To enable easy implementation of the described QA protocol at other research facilities, all data and source code are available at [33].

### 2.5 Array noise correlation and geometry factor

An important performance metric of coil arrays is their capability of parallel imaging. This is particularly relevant for hyperpolarized studies, where the main source of SNR degradation when using parallel imaging is non-ideal characteristics of the coil array [22], [34]. Consequently, array metrics relevant for parallel imaging, besides SNR, were also evaluated for the three coil arrays included in the study (Coils #4, #5 and #6). More precisely, noise correlation matrices and geometry factors (g-factors) were estimated.

While the noise correlation matrix is an absolute metric of array performance, the g-factor is dependent on both the coil and subject geometry, the sampling scheme, the acceleration method and the acceleration rate. A g-factor map provides an estimate of noise amplification in the parallel imaging reconstruction. A g-factor greater than one indicates noise amplification, typically caused by low sensitivity differences between coils for aliased pixels. In this study, g-factors were calculated based on uniform retrospective undersampling in a single suited direction and acceleration rates (R) of 2 and 4. Maps of the g-factor were generated based on the extracted coil sensitivity profiles as described in [35] using source code published by M. Hansen [36]. The maps were calculated similarly for all three arrays, with the only difference being the direction of the undersampling pattern: left–right for Coils #4 and #6, and anterior–posterior for Coil #5. This difference accounts for the different geometries of Coils #4 and #5 with respect to the sample.

## 3 Results

### 3.1 SNR – SAM phantom

**Fig. 4:**
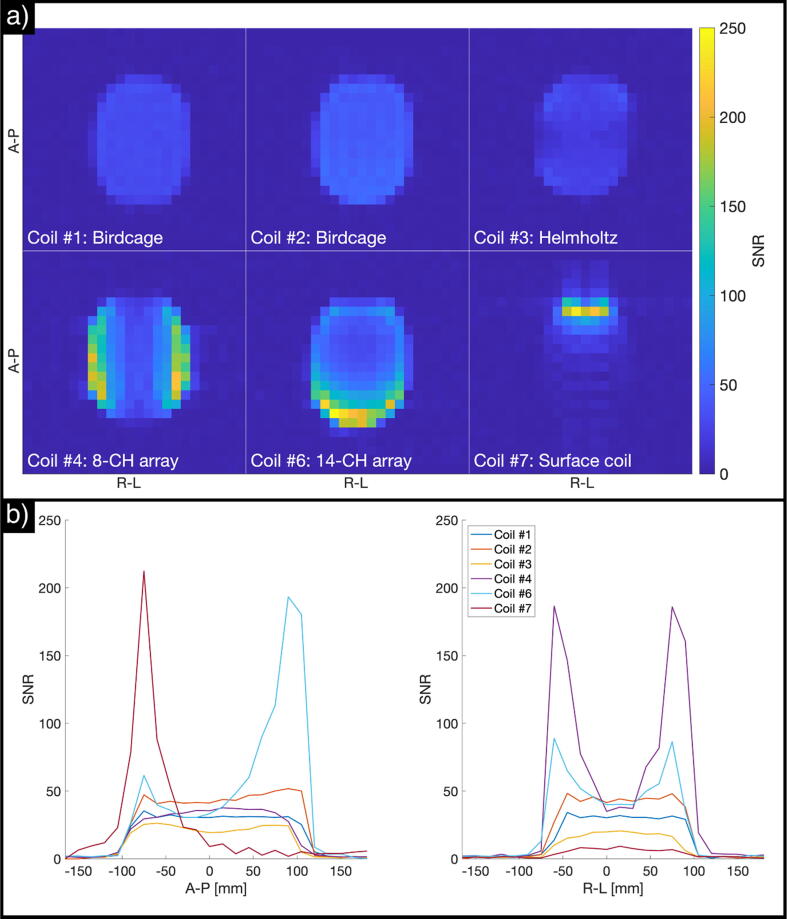
SNR estimates obtained with the SAM phantom. (a) SNR images, (b) SNR profiles across the central axes of the measured slice (anterior–posterior on the left side, right–left on the right side).

The SNR images measured using the SAM phantom for the different coils are shown in Fig. 4a. Fig. 4b shows SNR profiles as the average SNR across three central voxels for the directions anterior–posterior and right–left.

Table 2 summarizes the SNR results for center and surface values for each phantom for all coils. Coil #2 (birdcage) shows the highest SNR (43) at the center of the phantom. The SNR levels of the other volume coils (Coils #1 and #3) reach only 72% and 47%, respectively, of the center SNR for Coil #2. The SNR distributions are consistent with the coil geometries, and Coil #3 (the Helmholtz pair) has the least homogenous volume of the three volume coils. The arrays (Coils #4 and #6) show lower SNR levels at the center compared to Coil #2 (86% and 94%, respectively, of the center SNR for Coil #2). The relative SNR levels are easiest appreciated from the line-plots in Fig. 4b.

**Table 2:** Summary of SNR results for the evaluated coils. SNR center values are based on the average across the three-by-three central voxels within the phantom. SNR surface values represent the maximum SNR level measured for each coil.

	SAM phantom				Cylindrical phantom			
	SNR center		SNR surface		SNR center		SNR surface	
	Absolute	Relative	Absolute	Relative	Absolute	Relative	Absolute	Relative
Coil #1	31	72%	36	14%	25	91%	35	11%
Coil #2	43	100%	56	22%	–	–	–	–
Coil #3	20	47%	39	15%	8	28%	30	9%
Coil #4	37	86%	214	85%	13	46%	161	49%
Coil #5	–	–	–	–	12	43%	189	57%
Coil #6	40	94%	252	100%	28	100%	330	100%
Coil #7	8	19%	238	94%	8	29%	249	75%

As expected, surface SNR is clearly higher for the arrays compared to the volume coils, reaching levels > 200 (roughly four times higher than Coil #2) at the very surface of the phantom. The exact surface SNR values are subject to some variation, both due to poor anatomical fitting of the arrays to the head-shaped SAM phantom and due to variations in slice location relative to the center of the array loops. Nevertheless, the absolute values obtained here are similar.

The results from the transmit map measurements for Coil #2 (birdcage) and the transmit coil used with Coils #4–7 (clamshell) are shown in Fig. S1 in the supplementary material and reveal a more homogeneous transmit map for the birdcage coil, compared to the clamshell coil. The mean and standard deviation of the flip angles relative to the nominal were 1.07 ± 0.07 for the birdcage and 0.99 ± 0.28 for the clamshell. Based on the transmit maps, a T_1_ estimate of 700 ms and a steady-state assumption, the theoretical SNR impact of the transmit inhomogeneity was also estimated. The estimation predicted less than 1% average SNR loss for Coil #2 and less than 8% for the clamshell coil. Maximum SNR losses were estimated at less than 3% for Coil #2 and less than 23% for the clamshell coil. The average and maximum values were calculated based on all phantom voxels in Fig. S1b and d.

The estimated T_2_* relaxation times had a mean value of 88.5 ms across the six SAM phantom measurements and a standard deviation of 11.0 ms. The relatively large variation is mainly caused by a bad shim for the Coil #7 measurement, which led to a T_2_* of 68 ms. The mean and standard deviation for the remaining five SAM measurements were 93 ± 5 ms.

### 3.2 SNR – Cylindrical phantom

**Fig. 5:**
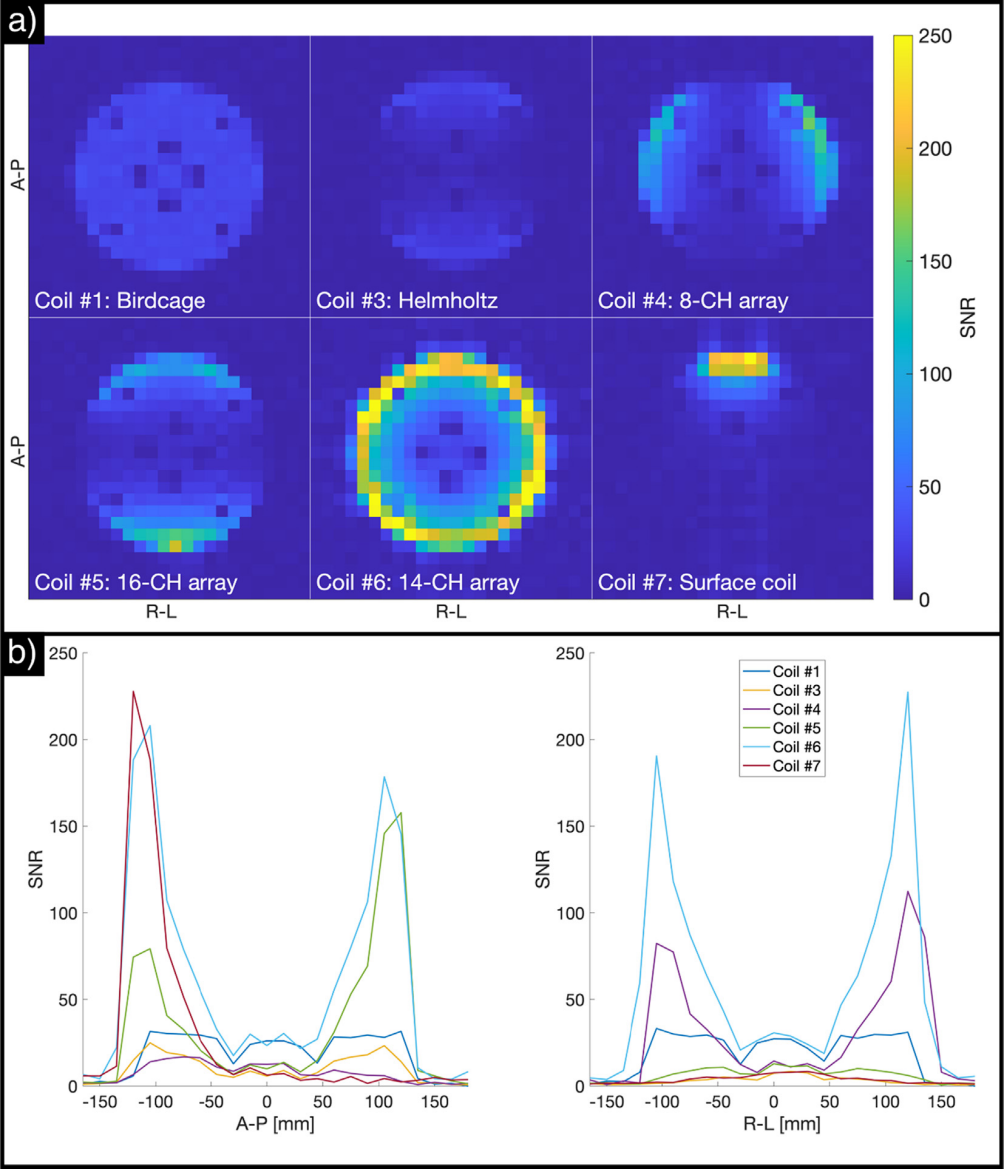
SNR estimates obtained with the cylindrical phantom. (a) SNR images, (b) SNR profiles across the central axes of the measured slice (anterior–posterior on the left side, right–left on the right side).

The estimated SNR images and profiles using the cylindrical phantom for the different coils are shown in Fig. 5.

Since Coil #2 was not measured with the cylindrical phantom, the highest center SNR in this case is 28 obtained with Coil #6. Center SNR is similar to that measured for the SAM phantom for the coils with rigid geometry (Coils #1 and #6), and worse for the coils that have some degree of mechanical flexibility (Coils #3, #4 and #7). This is expected, as the surface-to-center distance of the cylindrical phantom is greater (125 mm) than that of the SAM phantom (~100 mm).

The maximum surface SNR levels for Coils #3 and #4 are lower with this phantom: 30 vs. 39 for Coil #3, and 161 vs. 214 for Coil #4. This is most likely due to the loading of the coils for each of the phantom setups. Due to the inherently low sample loading at the ^13^C frequency, most of the loading experienced by these coils comes from their own mutual coupling when their two parts are opposed. Therefore, this type of coil has to be designed such that there is an optimal distance between their moveable parts, which in this case seems to be the size of a human head.

The estimated T_2_* relaxation time constants were 79 ± 3 ms across the six cylindrical phantom measurements.

### 3.3 Array noise correlation and geometry factor

**Fig. 6:**
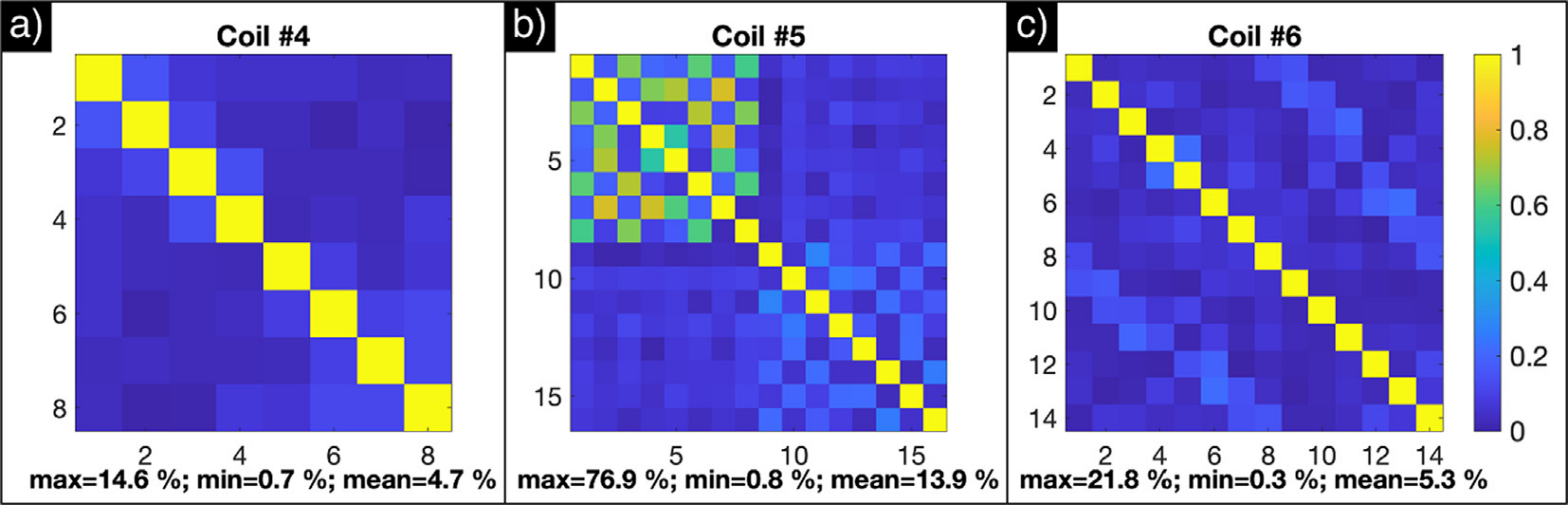
Coil noise correlation for the arrays used in this study: (a) Coil #4 (8-channel array), (b) Coil #5 (16-channel array) and (c) Coil #6 (14-channel array).

The noise correlation matrices of the arrays are shown in Fig. 6. The results originate from the cylindrical phantom acquisitions and show that Coils #4 and #6 perform similarly in terms of maximum and mean correlation between elements, with relatively low maximum correlation factors of 15% and 22%, respectively. However, the top part of Coil #5 (element 1–8) shows elevated correlation factors, with a maximum correlation > 70%. Since the noise correlations were included in the image reconstruction (Eq. (2)), the SNR penalty expected from high noise correlations were partially compensated for the top part of the array. This is demonstrated by Fig. S2 in the supplemental material, which shows SNR images for Coil #5 reconstructed with and without inclusion of the noise correlations. By including the noise correlations, ~1.5 times higher SNR levels were obtained for the top part.

**Fig. 7:**
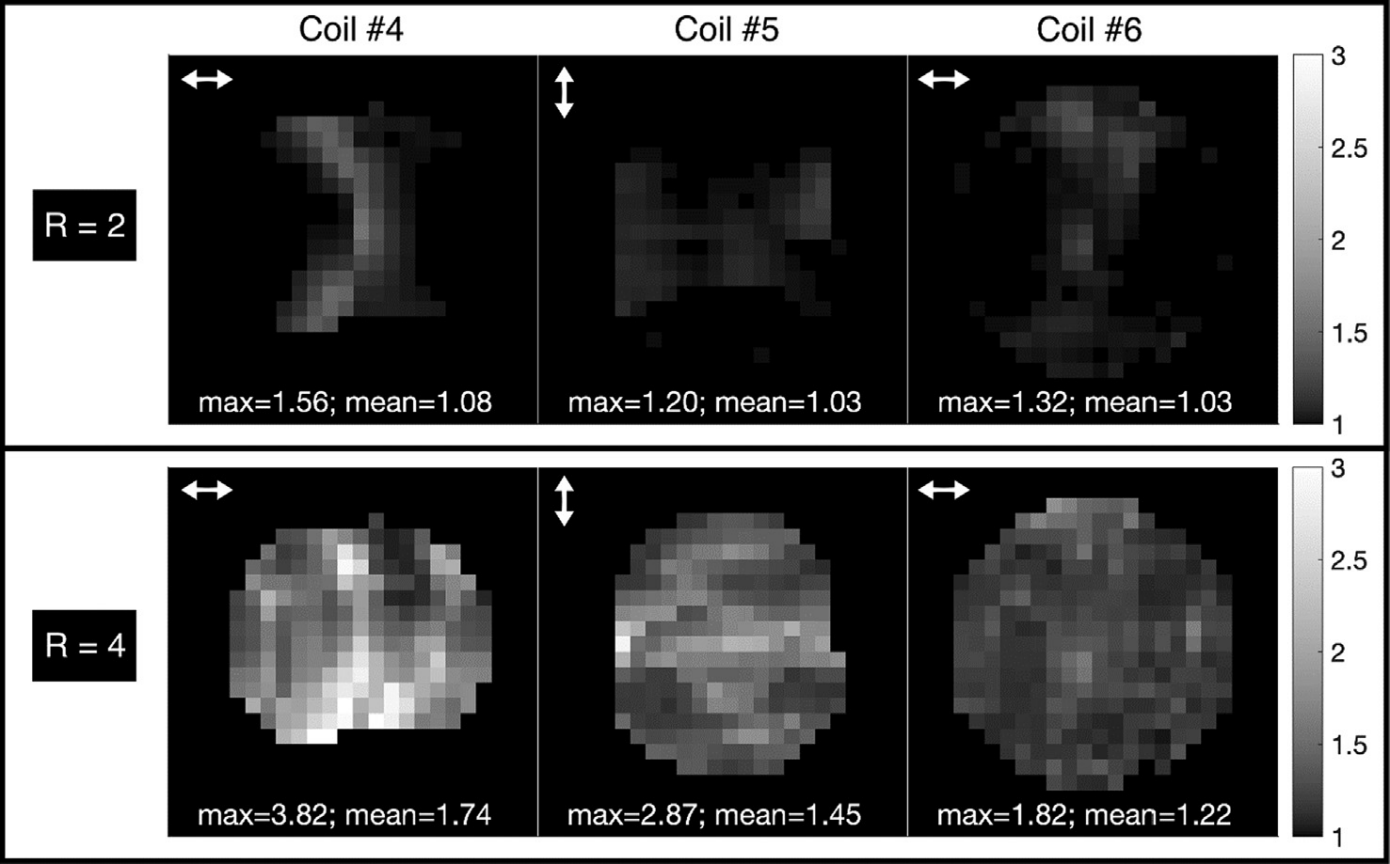
Parallel imaging g-factor maps for two acceleration factors, R = 2 and R = 4 (directions indicated by arrows) for the arrays used in this study: Coil #4 (8-channel array), Coil #5 (16-channel array) and Coil #6 (14-channel array).

The estimated g-factor maps for undersampling with acceleration rates R = 2 and R = 4 are shown in Fig. 7. For R = 2, the obtained g-factors are overall low and similar for all three arrays. For the higher acceleration rate, R = 4, the difference between the 8-channel and the other arrays becomes more significant, showing potential for higher acceleration rates for the arrays with more elements. The g-factors are lower for Coil #6 than for Coil #5 despite the fewer elements of Coil #6 (14 vs. 16 elements).

## 4 Discussion

MR receive coils for ^13^C at 3T are a crucial part of the hardware needed for hyperpolarized ^13^C experiments. Routine quality assurance is therefore important to ensure optimal performance of available coils and to evaluate new coil developments. The proposed QA protocol allows coil evaluation across coil types and scanner vendors, demonstrated by the comparison of seven different RF receive coils.

The SNR results presented here show great variability in the performance of the three evaluated volume coils with up to 115% difference in SNR at the coil center for the human head phantom. For the two volume coils with similar geometry (birdcage coils, Coil #1 and #2), Coil #2 showed ~1.4 times higher SNR performance compared to Coil #1. While Coil #2 is slightly smaller than Coil #1 and the field strength of the GE scanner in Cambridge is 3.7% higher than that of the Siemens scanner in Copenhagen, this does not fully explain such large difference in performance. Factors such as protection circuits and dual-tuning cannot be assessed. It is possible that a small frequency shift (detuning) of Coil #1, for the high-Q and low sample loading at this frequency, could have a significant impact on its performance. Furthermore, the fact that ^13^C volume coils cannot be shielded (because they need to be transparent to the ^1^H signal) makes them more sensitive to coupling to the scanner bore, which could also contribute to imperfect tuning and matching.

The results of the evaluated arrays also showed some SNR differences, but were in general as expected, especially with respect to surface SNR. The comparison to the single-element receive loop showed that the performance of all the arrays was at least 64% of the performance of the single coil. The performance of the home-built array, Coil #6, demonstrated an array configuration that can both reach a high SNR level at the coil surface (compared to a single element), and that has limited SNR loss furthest away from the coil surface (28% with respect to the best volume coil). The SNR results of Coil #5 demonstrated a case of high noise correlations and showed that inclusion of the noise correlation matrix in the reconstruction has a significant impact on SNR, when correlations between elements are high.

The results obtained for the two phantoms were similar for rigid geometry coils, despite the smaller volume of the SAM phantom; nearly half the volume of the cylindrical phantom. This suggests a negligible effect from sample loading at this frequency [37].

The comparison of volume and surface coils showed SNR performance of the arrays that consistently outperformed the SNR of the volume coils for the first 7 cm depth from the phantom surface [26]. The volume coils only proved superior at deeper locations. For a good-performing coil array, the SNR sacrifice at deep locations (e.g. at 11 cm) is less than 25% in this study. This SNR loss should be put in context of the parallel imaging capability of the arrays, giving them a performance advantage with respect to encoding efficiency. On the other hand, the volume coils have the benefit of higher receive homogeneity, which results in more readily interpretable images. In order to produce uniform sensitivity images for coil arrays, the coil profiles are needed. Coil profile estimation has been shown possible based on simulations and phantom measurements for rigid geometry coils at this frequency due to the aforementioned negligible sample loading [37], [38]. For coil combination using simulated or phantom estimated coil profiles to be SNR-optimal, however, the profiles need to be accurately registered to the hyperpolarized image acquisition. For this purpose, coil markers and a robust registration procedure are required. If uniform sensitivity images are not needed, the combination method presented by Zhu et al. can be used without the need for coil markers [15]. The relative performance between Coils #2 and #4 reported here is consistent with published data obtained with similar coils [39].

While the focus of this study has been on receive coil performance, the presented SNR results cannot be viewed independently of the performance of the transmit coil. Getting more independent measurements of receive coil performance would require a transmit map measurement in connection with each SNR acquisition and would prolong the total scan time significantly, which would be problematic for regular quality assurance. Additionally, a receive-only coil would never be used without a transmit coil, and evaluating it independently of such would hence not reflect actual use.

The transmit-only coil used together with the receive-only coils included in this study (the clamshell coil), is currently the only transmit-only coil available for human hyperpolarized ^13^C MRI, and the study hereby reflects actual use for the tested receive-only coils (Coils #4–7). Fig. S1 in the supplementary material shows the relative FOV locations for the acquisitions using the clamshell coil and demonstrate that approx. 10% local SNR improvements could be obtained if a more homogeneous transmit coil had been used. This analysis estimates a “worst-case-scenario”, with the phantom positioned at the bottom of the clamshell, while the homogeneous part of the clamshell is centered ~5 cm above the scanner isocenter [39]. Due to this relatively low SNR loss of approx. 10%, we consider that SNR maps without B1+ correction, still provide a good estimate of receive coil performance.

The SNR estimates for the surface coils are subject to a higher degree of uncertainty compared to the rigid volume coils. This is both due to effects from exact slice location relative to the center of the coil loops and because these all used the more inhomogeneous clamshell coil for RF transmission. Up to 10% variation in SNR values are expected for day-to-day measurements. A higher degree of repeatability could be assured with coil markers, fixed coil and phantom positioning and a more homogeneous transmit coil, but for routine assessments of overall coil function, we do not see this as a necessity. For incremental improvements with respect to coil developments, on the other hands, a high repeatability is critical. *For the GE system in Aarhus, where most of the data were acquired, variations in center frequency and transmit power calibrations within repeated measurements were less than 5%.*

The exact level of repeatability for the QA protocol was not assessed in this study, but a general expectancy can be based on Fig. S3 in the supplemental material. The figure shows the SNR estimation for Coil #5 with the cylindrical phantom together with a repeated measurement, with the only intentional difference being higher order shimming instead of automatic linear shimming. The SNR maps are largely similar, but the measurement preceded by higher order shimming has overall higher SNR (18% higher). This is not surprising given the large discrepancy in field homogeneity with a mean T_2_* value of 82 ms for the automatic shimming measurement and a mean T_2_* of 132 ms for the higher order shimming measurement. As described in the QA protocol, signal values for the SNR calculations were extracted as the first in-phase time points instead of maximum peak amplitudes to mitigate effects from varying field conditions. And although the effects were not mitigated completely for this large homogeneity difference, the relative difference in SNR between the two measurements is still less compared to the relative SNR difference calculated based on peak amplitude (31%, also shown in Fig. S3).

For the evaluation of the array coils’ parallel imaging capabilities, Coil #6, the home-built array, showed the most promising results with low g-factors at both acceleration factors 2 and 4. Coil #5 had slightly higher g-factors at R = 4, despite the higher number of elements. However, taking the arrangement of the coil elements into account, the results make sense: Coil #5 has two rows of 8 elements each in the through-plane direction, while the 14 elements of Coil #6 are all arranged in a single row. Therefore Coil #5 would also allow for undersampling along the through-plane axis, not possible for Coil #6. For volume acquisition with a uniform undersampling pattern, Coil #5 is therefore expected to show superior parallel imaging performance (compared to the single-slice acquisition used here).

Minor variations of the proposed QA protocol were necessary during acquisition prescription for the Siemens MR system. These included the spectral bandwidth (10,000 Hz vs. 5000 Hz) and the number of acquired points (2048 vs. 1024) for both the CSI and noise measurements. Additionally, the Siemens noise measurement was acquired as a CSI with matrix size 8x8, however without any RF power. SNR was in this case calculated based on noise evaluations for all 2048x8x8 data points. Such minor variations should not affect coil comparisons, as long as any changes in bandwidth per point are compensated by simple scaling with the square root of the sampling period.

Altogether, the results presented here demonstrated the importance of performing careful evaluations of ^13^C coils. This promotes the purpose of the proposed QA protocol as a reliable method to ensure well-functioning receive coils and aid coil developments for sites performing ^13^C MR. A critical feature of the proposed protocol is the coil combination for coil arrays, which allows comparison of volume coils and arrays with different numbers of elements.

## 5 Conclusion

In this study, we proposed a detailed protocol for QA evaluation of RF coils, which allows for sensible SNR calculation and coil combination, such that a fair comparison between volume and surface coils (with different channel count) can be made. This protocol has been applied to several dedicated ^13^C coil setups, showing substantial differences in performance. The different coils were compared in terms of SNR and parallel imaging capability. The results obtained show the importance of careful quality assurance of coils at this frequency.

The methods and results presented here will help the community of researchers and clinicians performing ^13^C experiments to choose or design the most appropriate coil for their experiment, depending on the depth of the target organ or structure from the coil surface and whether the planned FOV and resolution require the use of parallel imaging.

## Declaration of Competing Interest

The authors declare that they have no known competing financial interests or personal relationships that could have appeared to influence the work reported in this paper.
